# Supplementary material for: Content-rich biological network constructed by mining PubMed abstracts
Source: BMC Bioinformatics. 2004 Oct 8;5:147. doi: 10.1186/1471-2105-5-147 (PMC528731; doi:10.1186/1471-2105-5-147)
Supplement: Additional File 5 — The original Chilibot query results of the term "long-term potentiation (LTP)" and 22 other terms, limiting the latest references analyzed to the years 1990, 1995, 2000, and 2004. [file 1471-2105-5-147-S5.bz2 › chilibotAdditionalFile5/ltp1995/html/SYNAPTOTAGMIN_SYNAPTOPHYSIN.html]

 


 **SYNAPTOTAGMIN** and **SYNAPTOPHYSIN** 
  
Found 22 abstracts in PubMed,  **22 abstracts were retrieved and analyzed**.  


---

 Search Google  |
 PDF files only 
|  EDU domain only 

---

**Interactive relationship** (e.g. stimulation, inhibition, etc)

- Finally, subcellular fractionation and antibody bead purification demonstrate that  **synaptotagmin**  co purifies with  **synaptophysin**  and other synaptic vesicle markers in brain.  Ref: 1985919 J Biol Chem, 1991
- However, in the adrenal medulla,  **synaptotagmin**  was found in both  **synaptophysin**  containing microvesicles and in chromaffin granules that are devoid of  **synaptophysin** , suggesting a shared role for  **synaptotagmin**  in the exocytosis of small synaptic vesicles and large dense core catecholaminergic vesicles.  Ref: 1985919 J Biol Chem, 1991
- **Synaptotagmin**  bead immunoisolated organelles from these tissues primarily consisted of SVs but contained occasionally larger structures reminiscent of LDCVs that were absent from vesicle populations immunoisolated with a  **synaptophysin**  antibody.  Ref: 8366350 J Neurosci, 1993

**Parallel relationship** (e.g. studied together, co-existance, homology, etc.)

- Evidence is mounting that the vesicle proteins vamp, rab3A,  **synaptophysin** ,  **synaptotagmin**  and SV2 play an important role in regulated exocytosis, by regulating neurotransmitter uptake, vesicle targeting and fusion with the presynaptic plasma membrane.  Ref: 8144706 J Cell Sci Suppl, 1993
- Antibodies raised against the cytoplasmic domains of five of these proteins, vamp, rab3A,  **synaptophysin** ,  **synaptotagmin** , and SV2, were used to investigate their function.  Ref: 8422678 Cell, 1993
- The synaptic vesicle proteins SV2,  **synaptotagmin**  and  **synaptophysin**  are sorted to separate cellular compartments in CHO fibroblasts.  Ref: 7901222 J Cell Biol, 1993
- To determine whether GluT4 vesicles are related to synaptic vesicles, rat adipocyte low density microsomes LDM, which are rich in GluT4 vesicles, were screened for the synaptic vesicle proteins  **synaptotagmin** ,  **synaptophysin** , SV2, p29, rab3, and VAMP synaptobrevin by immunoblotting.  Ref: 1601842 J Biol Chem, 1992
- The membrane composition of nerve terminal derived coated vesicles was very similar to that of synaptic vesicles, containing the membrane proteins  **synaptophysin** ,  **synaptotagmin** , p29, synaptobrevin and the 116 kD subunit of the vacuolar proton pump, in similar stoichiometric ratios.  Ref: 1325974 J Cell Biol, 1992
- We expressed the synaptic vesicle proteins SV2,  **synaptotagmin** , and  **synaptophysin**  in CHO fibroblasts to investigate the targeting information contained by each protein.  Ref: 7901222 J Cell Biol, 1993
- Membrane proteins of SSV involved in fusion  **synaptotagmin**  and trafficking Rab 3 together with  **synaptophysin**  have been identified on SLMV.  Ref: 8520460 Neurochem Int, 1995
- Evidence is mounting that the vesicle proteins vamp, rab3A,  **synaptophysin** ,  **synaptotagmin**  and SV2 play an important role in regulated exocytosis, by regulating neurotransmitter uptake, vesicle targeting and fusion with the presynaptic plasma membrane.  Ref: 7568899 Prog Brain Res, 1995
- High magnification revealed that  **synaptotagmin**  I like immunoreactivity was mainly distributed in a fine granular pattern, but large, brightly fluorescent granules which were not labelled by anti  **synaptophysin**  or anti synapsin I were occasionally observed.  Ref: 7534885 Neuroscience, 1994
- The contrasting effects of neuronotypic differentiation on levels of  **synaptophysin**  and p65  **synaptotagmin**  indicate potential differences in the regulation of these proteins in PC12 cells.  Ref: 8380434 J Neurochem, 1993
- Immunoisolation of small and large vesicles from the sucrose gradient confirmed the differential distribution of  **synaptotagmin**  and  **synaptophysin**  in the two types of secretory vesicles in nerve endings of the posterior pituitary.  Ref: 8366350 J Neurosci, 1993
- KIF1A was associated with organelles that contained synaptic vesicle proteins such as  **synaptotagmin** ,  **synaptophysin** , and Rab3A.  Ref: 7539720 Cell, 1995
- We also created cell lines expressing both SV2 and  **synaptotagmin** , both  **synaptotagmin**  and  **synaptophysin** , and lines expressing all three synaptic vesicle proteins.  Ref: 7901222 J Cell Biol, 1993
- In particular, mRNAs encoding  **synaptophysin**  IIb and  **synaptotagmin**  II which is undetectable at E9 become predominant.  Ref: 7496630 Mol Cell Neurosci, 1995
- No  **synaptotagmin**  I like immunoreactivity was found in large axons, while accumulations of the synaptic vesicle proteins  **synaptophysin**  and synapsin I were found in all types of axons.  Ref: 7534885 Neuroscience, 1994
- To explore further the role of the presynaptic terminal in long term potentiation, we have investigated changes in three synaptic vesicle proteins, synapsin,  **synaptotagmin**  and  **synaptophysin** , in control tissue and in tissue prepared from potentiated dentate gyrus 45 min and 3 h after induction of long term potentiation.  Ref: 7914357 Neuroscience, 1994
- Several synaptic vesicle proteins including  **synaptophysin**  and p65  **synaptotagmin**  are expressed by the pheochromocytoma cell line PC12.  Ref: 8380434 J Neurochem, 1993
- These synapses expressed proteins characteristic of mature synapses immunofluorescence staining showed the presence of  **synaptophysin** ,  **synaptotagmin** , VAMP synaptobrevin 2, syntaxin and neurexin.  Ref: 8520575 J Physiol Paris, 1995
